# Postauricular Reconstruction With Multiple Dermal Regeneration Templates: A Case Report

**DOI:** 10.7759/cureus.107779

**Published:** 2026-04-27

**Authors:** Christos N Noulas, Markos A Markou, Georgios I Voulgaris, Nikolaos A Papadopulos

**Affiliations:** 1 Department of Plastic Surgery and Burns, University Hospital of Alexandroupolis, Alexandroupolis, GRC; 2 First Surgical Department, University Hospital of Alexandroupolis, Alexandroupolis, GRC

**Keywords:** dermal regeneration templates, drt, ear reconstruction, matriderm, ­skin cancer

## Abstract

Reconstructing postauricular and ear defects after skin cancer removal in elderly patients can be puzzling. This case report highlights Matriderm® as an effective and unconventional solution for addressing this difficult problem. The application of dermal regeneration templates (DRTs) in resource-limited settings and scenarios expands perspectives in surgical literature. Their usage not only addresses clinical challenges but also contributes significantly to surgical innovation and comprehension.

## Introduction

Basal cell carcinoma (BCC) is a variant of non-melanoma skin cancer, characterized by a high global prevalence ranging from 114 to 726 per 100,000, depending on geographical location. Patients with BCC are commonly elderly, have generally low-grade Fitzpatrick skin type, and are usually exposed to ultraviolet radiation. BCC is considered a manageable form of cancer, accounting for only 0.1% to 2% of all cancer-related fatalities among patients [1,2]. Skin cancer in the auricular region accounts for 6% of skin cancer located in the head and neck [3].

BCC usually arises on sun-exposed skin and is typically found in the neck and head region. It originates in the basal cell layer of the epidermis, where mutagenesis and thymine dimers are generated when UV rays damage DNA. For those who are unable to undergo surgery, a variety of therapeutic options are available, such as the topical use of 5-fluorouracil or imiquimod and medications such as vismodegib [1,2,4]. As surgical excision is the most efficient and affordable treatment for BCC, it is regarded as the preferred approach. However, as tumors need to be excised with adequate margins, the reconstruction can be challenging, frequently causing soft-tissue defects and scarring that may not be aesthetically satisfactory. Due to the possibility of tumor recurrence, reconstruction after ear and postauricular excisions can be challenging and includes tissue expansion, flaps, and skin grafting [5,6].

Dermal regeneration templates (DRTs) have gained widespread application in clinical settings for the management of wounds, burns, and reconstructive procedures [7]. Matriderm® is a dermal substitute consisting of a highly porous membrane constructed of collagen and elastin, derived from the bovine dermis and nuchal ligament [8]. Matriderm® offers a scaffold that supports fibroblasts, stimulating neovascularization and cellular migration. Succeeding the embedding of a DRT, skin redevelopment progresses via the following four distinct histological stages: scaffold imbibition, fibroblast migration, neovascularization, and remodeling with maturation. After collagen production by fibroblasts, Matriderm® dissolves over a six-week interval, resulting in skin regeneration and scar modulation, thereby reducing the likelihood of hematomas and infections [9]. This case study presents a complex scenario involving a postauricular and ear skin defect successfully reconstructed using the Matriderm® dermal substitute. To our knowledge, no other such cases have been reported in the literature.

## Case presentation

Our plastic surgery outpatient clinic received a referral for an 83-year-old man in poor health, with a history of diabetes mellitus and heart failure, diagnosed with BCC located in the postauricular, helix, and anthelix region. The patient had already undergone head and neck CT staging, which showed no evidence of metastasis or lymphadenopathy. An excisional biopsy was subsequently performed, which verified the previously stated nodular-type BCC with a depth of 5 mm. Clear and sufficient margins (3-7 mm) were attained. A split-thickness skin graft was used to cover the defect, and it healed fairly well.

**Figure 1:**
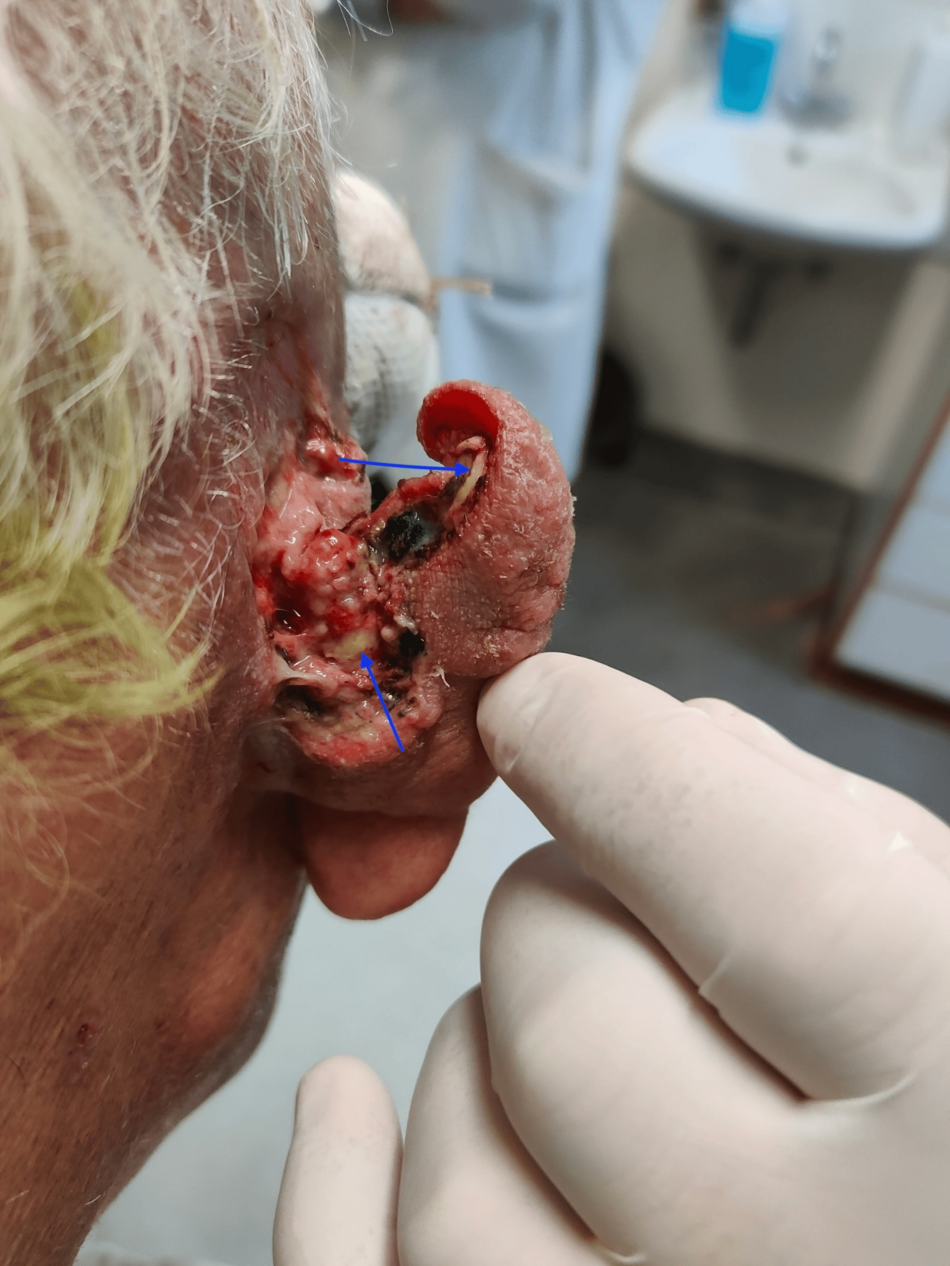
Post-excision defect of the right ear. Post-excision image of the right ear following wider excision for recurrent basal cell carcinoma. The image demonstrates a complex 4 × 3 cm defect involving the postauricular region, helix, and anthelix, with exposed helix cartilage following perichondrium removal (blue arrows).

Concerns about the graft’s appearance prompted the patient’s referral back to the outpatient clinic a year later. Specifically, an ulcerated area of 0.5 cm and redness, without pain or discharge, was observed. Consequently, BCC recurrence was clinically suspected. A wider excision was then performed in which the perichondrium was also removed, and the helix cartilage was exposed, resulting in a 4 × 3 cm complex defect (Figure 1).

As a reconstruction option, the application of four Matriderm® DRTs at different levels was promoted. Matriderm® was chosen due to its ability to support vascularization, because of the patient’s health condition, and his personal wish not to undergo a major reconstructive surgery, as he had no aesthetic demands. His main wish was to be discharged as soon as possible and return to his daily life. The fixation of Matriderm® to the cartilage required careful placement to ensure adherence; the Matriderm® was shaped appropriately, and securing sutures (3-0 Nylon) were placed in the cartilage and the surrounding skin.

**Figure 2:**
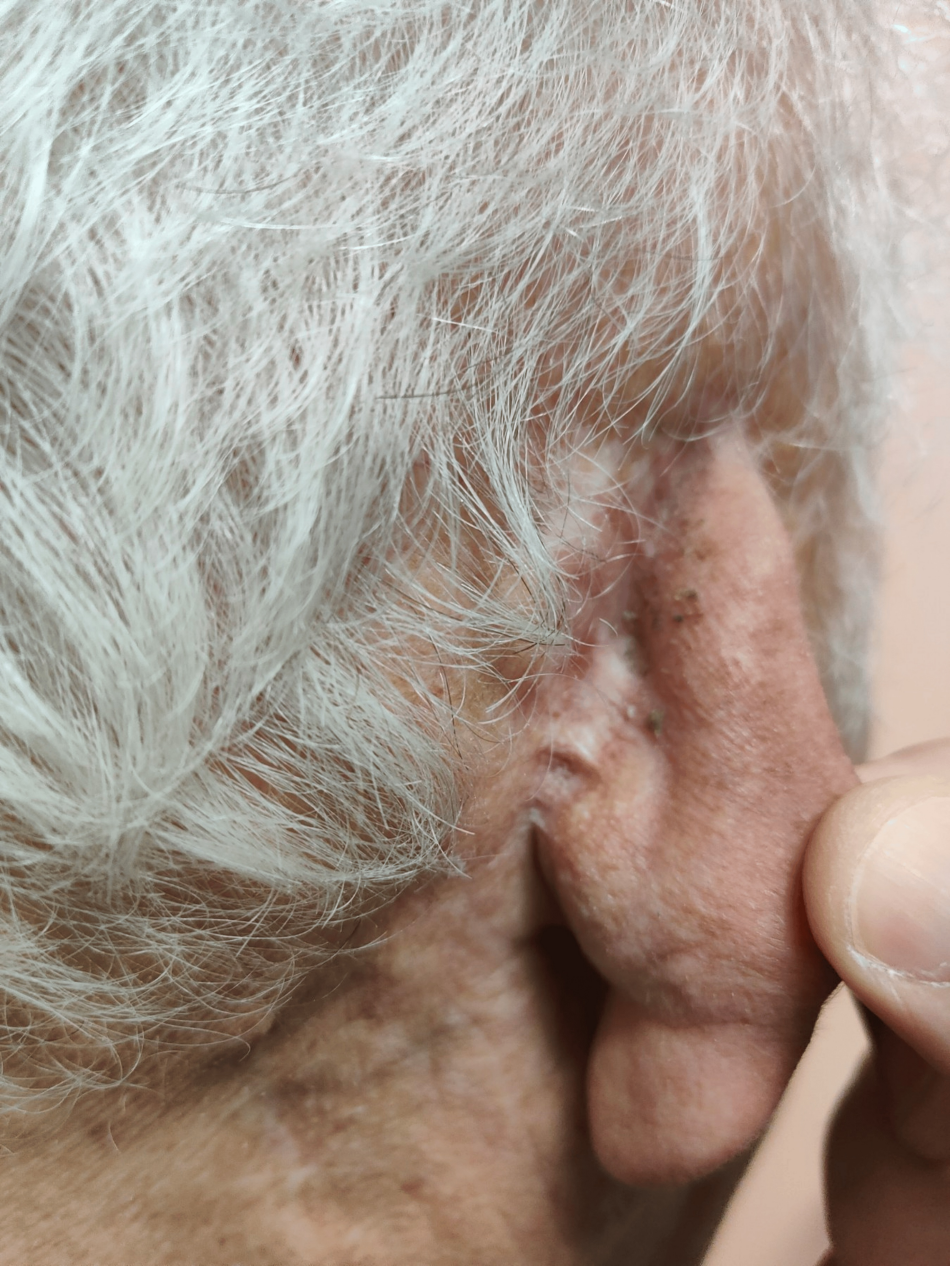
Four-month postoperative result of the right ear. Final outcome of the right ear four months after Matriderm® dermal regeneration template application. Complete epithelialization was achieved with satisfactory restoration of the anatomical contour of the helix and no signs of inflammation or recurrence.

Four months after frequent wound care at the outpatient clinic, the defect appeared fully epithelialized, innervated, and aesthetically satisfactory, given the patient’s age (Figure 2).

The wound care protocol was structured as follows: for the initial week, the Matriderm® dermal substitute was left undisturbed without dressing changes. Then, for six weeks, the patient underwent dressing changes three to four times per week, utilizing normal saline for irrigation and paraffin gauze for coverage. Subsequently, the frequency of dressing changes was reduced to twice per week until complete healing was achieved. Notably, complete integration of the Matriderm® was evident at the one-month follow-up, with full epithelialization accomplished by the fourth month. Throughout the healing process, no signs of inflammation were detected. The anatomical contour of the helix was restored to a satisfactory degree, and the patient expressed satisfaction with the outcome.

Following complete epithelialization at the fourth month, the patient was enrolled in a structured follow-up protocol, consisting of monthly outpatient clinic visits for the first year, followed by bimonthly visits for an additional year. No signs of recurrence were detected during this period.

## Discussion

Preservation of the external ear is not only a matter of cosmesis but also of function, as the external ear guides sound waves into the external acoustic meatus [3]. Postauricular reconstruction after the excision of malignancies can be particularly demanding [10]. The most common skin cancer types are BCC, squamous cell carcinoma, and, very rarely, melanoma [11]. The unique form of the external ear presents difficulties in reconstruction, particularly in elderly individuals, demanding thorough planning and advanced surgical skills [12].

The reconstruction method of the ear and postauricular defects is selected after considering variables, such as the defect's location, the health status of the patient, and the aesthetic outcomes. There are no standardized guidelines for postauricular reconstruction, as multiple surgical options may be appropriate depending on the clinical context. Available techniques include primary closure, secondary healing, skin grafts, tissue expanders, local or distant flaps, and ear prostheses [10,11,13].

When evaluating reconstruction options for complex ear defects, several factors must be considered. Primary closure is often not feasible for large defects, while skin grafts, although commonly used, require a vascularized wound bed and may yield suboptimal aesthetic results. Local and distant flaps, while effective, demand expertise, longer operative time, general anesthesia, and prolonged hospitalization, which may not be suitable for elderly patients with significant comorbidities. Tissue expanders require multiple surgeries, further limiting their applicability in high-risk patients. In this context, Matriderm® offered a valuable alternative: it can be applied under local anesthesia, requires no donor site, avoids the complications associated with flap surgery, and allows for early patient discharge and return to daily activities. These characteristics made it particularly suitable for our patient, who presented with diabetes mellitus, heart failure, and a strong preference for minimal surgical intervention.

Regarding complications of reconstructive ear surgery, the most common is bleeding during the operation. There have also been reports of a high incidence of postoperative infections such as perichondritis and external canal stenosis [6].

In our case, we were confronted with a highly complex defect of the posterior surface of the right ear, involving exposed helix cartilage following perichondrium removal, in an elderly patient with significant comorbidities. Additional resource limitations regarding available operating rooms were in place due to the COVID-19 pandemic. The patient had no significant aesthetic concerns apart from wishing to preserve his ear, while he also emphasized a strong desire for early discharge and return to daily activities. To our knowledge, this is the first reported case of ear reconstruction using multiple DRTs applied at different levels covering exposed cartilage, representing a novel application of this material in auricular surgery. The reconstruction was favored due to the simplicity of the method, the absence of donor site morbidity, and the possibility of early patient discharge. Similarly, our team’s previous report described scalp reconstruction in an elderly male with similar health restrictions and minimal aesthetic anticipations, where a full-thickness defect with periosteal removal was managed with Matriderm® as a salvage solution. In both cases, Matriderm® proved to be a direct and effective option in resource-limited settings, exhibiting its versatility in managing intricate defects involving perichondrial or periosteal removal and supporting swift recovery for patients.

## Conclusions

In resource-limited scenarios where a fast and decisive intervention needs to be taken, Matriderm® can help resolve complex reconstructive challenges. To our knowledge, ear reconstruction with multiple DRTs in ear deficits has not been reported in the literature. Although more complex reconstructive techniques are well-documented, they often require longer operative time and hospitalization, and resources are not always available. DRTs can potentially provide a simple and effective reconstruction solution for complex posterior ear defects following skin cancer excisions. Importantly, while complete epithelialization was achieved at four months, the simplicity of the outpatient wound care protocol allowed the patient to return to daily activities almost immediately after the procedure, without the need for prolonged hospitalization.
